# Foot rubbing evaluation of friction between shoe and flooring

**DOI:** 10.1371/journal.pone.0275385

**Published:** 2022-09-29

**Authors:** Kei Shibata, Akihiro Ohnishi

**Affiliations:** National Institute of Occupational Safety and Health, Kiyose, Tokyo, Japan; University of Belgrade: Univerzitet u Beogradu, SERBIA

## Abstract

A simple and inexpensive method to evaluate slip resistance that can be readily introduced into the workplace is required. In the present study, we investigated the relationship between a simple sensory evaluation of anti-slipperiness by foot rubbing in standing and sitting positions and the actual friction properties obtained with in situ measurements at slip onset and during sliding. We also verified the possibility of identifying a hazardous area with a high slip-induced fall risk by the sensory evaluation scores. At the foot rubbing tests, more than half of the 15 participants in experiments could adequately perceive the slip resistance using the proposed method without any education on its perception. Furthermore, hazard detection scores, where a friction coefficient of 0.2 was detected as hazardous area, were obtained from optimal cut-off points of receiver operatorating characteristic curves for the participants with friction perception capability. The scores were 28.7, 20.7, 24.7, and 52.3/100 for the slip onset while standing, sliding while standing, slip onset while sitting, and sliding while sitting, respectively. From the viewpoint of hazard detection accuracies, the standing position was a better way than the sitting although limited to participants with the capability of perceiving friction. Based on the analysis of how to apply forces, the participants who showed a small movement of the center of pressure while standing or an increase in the vertical load while sitting had the capability of perceiving friction.

## 1. Introduction

Slips, trips, and falls have attracted attention from Japanese companies and administrative organs in recent years because of the high rates of occupational injuries associated with them [1, 2]. In particular, slip-induced falls constitute one of the leading categories of non-traffic accidents in terms of serious injuries and fatalities, and are a primary cause of workplace injuries [3]. Even on a worldwide scale, the majority of falling accidents are caused by slips [4–7]. To reduce slip-induced falls at the workplace, we might first evaluate the slip resistance between a type of flooring and shoes using a tribometer. The coefficient of friction (COF) is often used to evaluate the slip resistances of flooring and footwear outsoles [8–13]. During walking, slip initiation is related to a static COF (SCOF), and slip continuity is related to a dynamic COF (DCOF). Both the SCOF and DCOF are important indicators that should be evaluated. Existing field-based (portable) tribometers possess meaningful reliability for measuring friction properties such as slip resistance. These include horizontal pull slipmeters [14], portable articulated strut tribometers [15], British portable skid testers [16], tortus devices [17], portable inclineable articulated strut tribometers [18], and cart-type friction measurement devices [19]. However, it is sometimes difficult to introduce tribometers into the workplace because of their portability, handleability, expense, and time costs. Therefore, a simple and inexpensive method to evaluate slip resistance that can be readily introduced into the workplace is required.

We believe that a sensory evaluation by people is better way to evaluate slip resistance if it is accurate. Li et al. [20] used a portable articulated strut tribometer to measure the COFs of five floor materials commonly used on a university campus under five surface conditions, including dry and four liquid spillage conditions. They found that Spearman’s rank correlation coefficients between the subjective (sensory) score and measured COF using neolite footwear were in the range of 0.8–0.975 for the five floors under all surface conditions. Courtney et al. [21] examined the association between the perception of slipperiness and risk of slipping in a 12-week prospective cohort study with 475 workers from 36 limited-service restaurants as participants. They concluded that each 1-point increase in the mean restaurant-level perception of slipperiness (on a 4-point scale) was associated with a 2.71-fold increase in the rate of slipping. Therefore, they suggested that safety professionals, risk managers, and employers could use aggregated worker perceptions of slipperiness to identify slipping hazards and potentially assess the effectiveness of intervention actions. Morio et al. [22] investigated the relationship between objective measurements of the available (or utilized) coefficient of friction and the subjective perception of grip or slipperiness in sport-like movements. They determined a threshold using probit models, which meant that below this threshold, the grip perception was not acceptable, whereas above this threshold, the grip felt good enough to perform the sport-like task. They concluded that strong relationships between subjective perceptions and objective measurements of friction were found in sport-like movements. Similar to these studies, many researchers have investigated the relationship between a sensory evaluation of anti-slipperiness and friction properties or fall risk. However, to the best of our knowledge, detailed research to determine a simple (easy to introduce into workplaces) sensory method that could find a hazardous area with a high slip-induced fall risk has not yet been conducted.

Thus, the first aim in the present study was investigation of the relationship between a simple sensory evaluation of anti-slipperiness by foot rubbing and actual friction properties obtained with in situ measurements of slip onset and during sliding. The second aim was verifying the possibility of identifying a hazardous area with a high risk of slip-induced falls by the foot rubbing method. For the foot rubbing tests, two test positions for each participant were proposed: standing and sitting. Foot rubbing in the standing position is a familiar way to check slip levels in everyday life. The disadvantage of the standing position is the need for safety considerations to avoid falling during foot rubbing. In contrast, foot rubbing in the sitting position is safer than that in the standing position. However, because most people have no experience with foot rubbing while sitting, they may feel strange. Additionally, it was assumed that the vertical force on the test sheet in the sitting position was smaller than that in the standing position. The third aim in this study was determination of the test position with superior accuracy.

## 2. Experimental details

### 2.1. Materials

**Fig 1:**
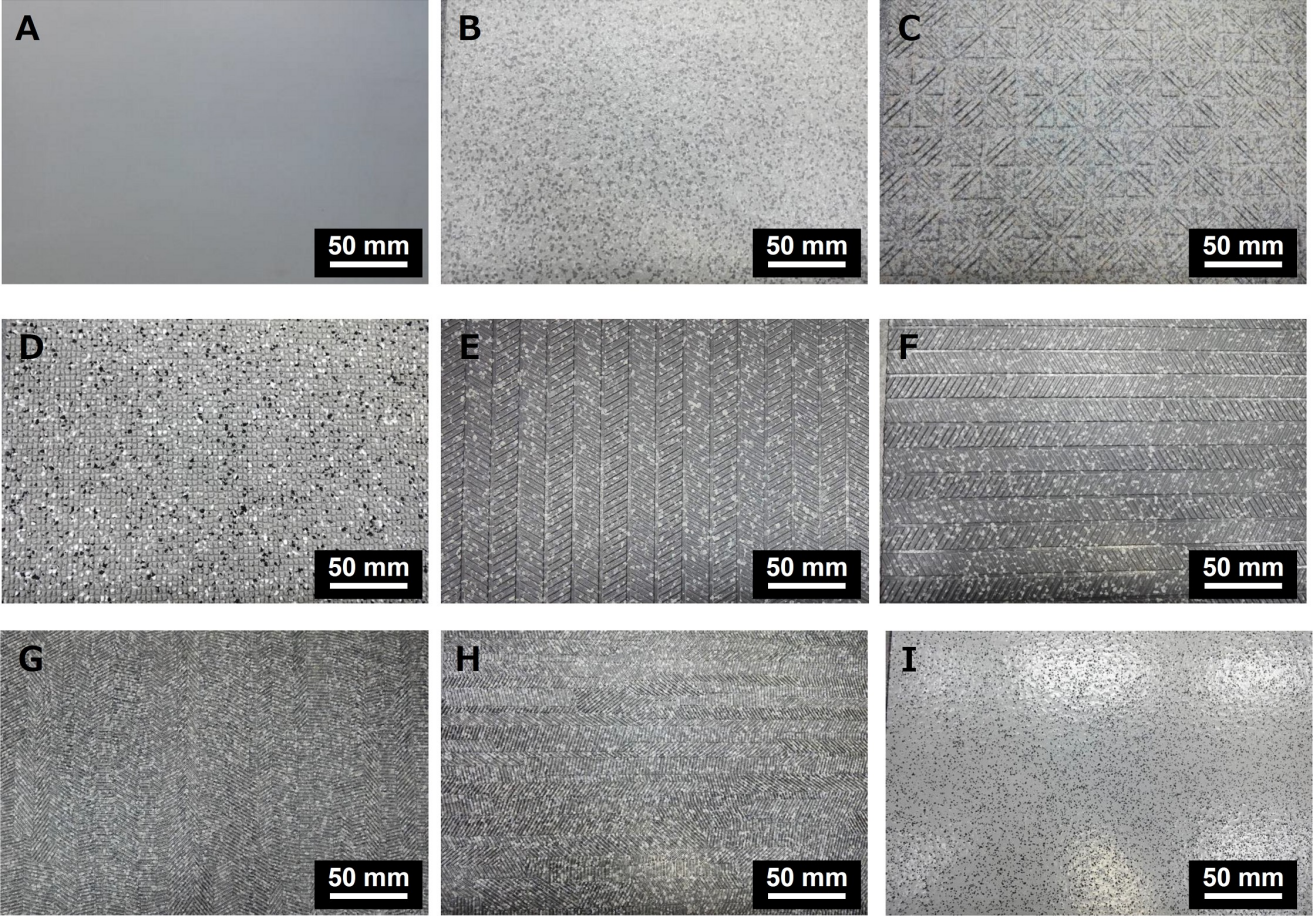
Photos of test sheet samples.

Fig 1 shows the photographs of test sheet samples. Nine commercially available sheets were selected for testing to determine the differences in their COFs. The dimensions of the sheets were 60 mm × 30 mm. Sheet A was a transparent 0.2 mm thick polypropylene film without surface patterns. Sheets B–I were made of polyvinyl chloride and were 2.5 mm thick. Sheet B had a smooth surface without a remarkable block pattern. Sheet C had a surface pattern that consisted of regularly arranged 1 mm tall triangular blocks and square blocks with rounded corners. Sheet D had grid-like grooves with a depth of approximately 1 mm. Sheet E had a parallelogram block pattern, with a height of 0.5 mm. The short sides of the parallelogram blocks were placed in the sliding direction. Sheet F was rotated 90° compared to sheet E. Sheet G had a tightly arranged half-cylinder pattern with a height of approximately 0.6 mm. Almost all the ends of the cylinders were headed in the sliding direction. Sheet H was rotated 90° relative to sheet G. Sheet I included hard particles on its surface. The durometer hardness (A/15) values of sheets B–I were 81, 83, 78, 86, 86, 82, 82, and 90, respectively. These durometer hardness values were just references because a thickness of more than 6.0 mm needs to measure durometer hardness.

**Fig 2:**
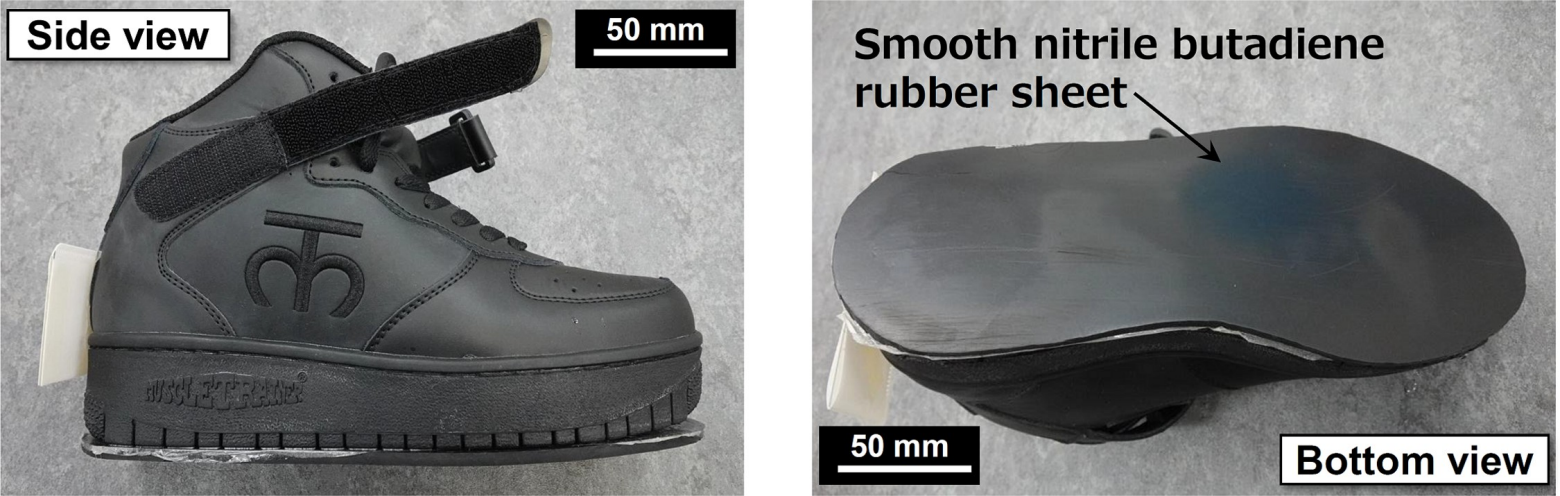
Photos of test shoe samples.

Fig 2 shows the photographs of test shoe samples. The shoe sample was a type of training footwear commercially available in Japan. This footwear was selected because it has almost no curvature in the shape of its sole and moderate weight. Four sizes of footwear were provided to fit the feet of the participants. The lengths of the shoes were 240–270 mm and their weights were 1.2–1.4 kg. A smooth nitrile butadiene rubber sheet was attached to the sole of the shoe to eliminate the effect of the tread block. Its thickness was 5 mm. A participant was asked to wear the footwear on both feet, whereas the smooth rubber sheet was only attached to the right foot, which was the test foot.

Prior to the main sensory tests, mechanical friction tests were conducted between the test sheets and test shoe. We used a cart-type friction measurement device [19] to measure the COFs. An experimenter pushed the device, and the shoe sample was dragged against the flooring using the device. The total load was 231 N, which included the masses of the jig and shoe samples. The total load was adjusted to match the critical load, which is the load that appears when a rapid slip occurs during walking and corresponds to 30% of the body mass [23]. The lubricant was a 90 wt% glycerol solution (136 mPa·s at 25°C), which was based on Japanese Industrial Standard T 8106:2020 (test method for slip resistance of protective and occupational footwear) [24]. The amount of solution was approximately 200 g/m^2^, and it was spread uniformly on the sheet sample. This amount was sufficient to cover the top surface. Three test replicates were used. The angle of test shoe against the test sheet was zero. The measurement results for the COFs are presented in Table 1. Here, the SCOF is the static coefficient of friction and the DCOF is the dynamic coefficient of friction at a sliding velocity of 0.3 m/s, which is based on the Japanese Industrial Standard [24]. As described later, dry and oil- (glycerol-solution-) lubricated stainless-steel vats were used as standard specimens. The test sheets had a wide range of COFs from 0.05 to 0.79.

**Table 1:** Mean SCOFs and DCOFs between test sheets and test shoe found in mechanical friction tests.

Sheet	Mean SCOF	(S.D.)	Mean DCOF	(S.D.)
A	0.049	0.025	0.102	0.036
B	0.423	0.013	0.313	0.021
C	0.202	0.046	0.314	0.045
D	0.117	0.016	0.185	0.043
E	0.344	0.056	0.217	0.014
F	0.229	0.034	0.211	0.048
G	0.289	0.049	0.343	0.020
H	0.309	0.058	0.462	0.009
I	0.583	0.090	0.546	0.056
Dry stainless-steel vat	0.778	0.144	0.786	0.172
Oil-lubricated stainless-steel vat	0.037	0.033	0.059	0.056

### 2.2. Participants

The study included 10 healthy adult males and 5 healthy adult females who did not have orthopedic disorders or musculoskeletal symptoms in their lower limbs. Their ages, heights, body weights, and shoe sizes were 40.0 ± 10.0 years, 1.71 ± 0.09 m, and 70.8 ± 14.0 kg, and 0.260 ± 0.015 m (mean ± standard deviation), respectively. The participants were informed of the protocol, and informed consent was obtained from each participant before the experiment. All the experimental procedures and protocols were approved by the Human Research Ethics Committee of the National Institute of Occupational Safety and Health, Japan, in 2021. None of the participants received any education or practice regarding the perception of slip before the test was conducted.

### 2.3. Experimental procedure

**Fig 3:**
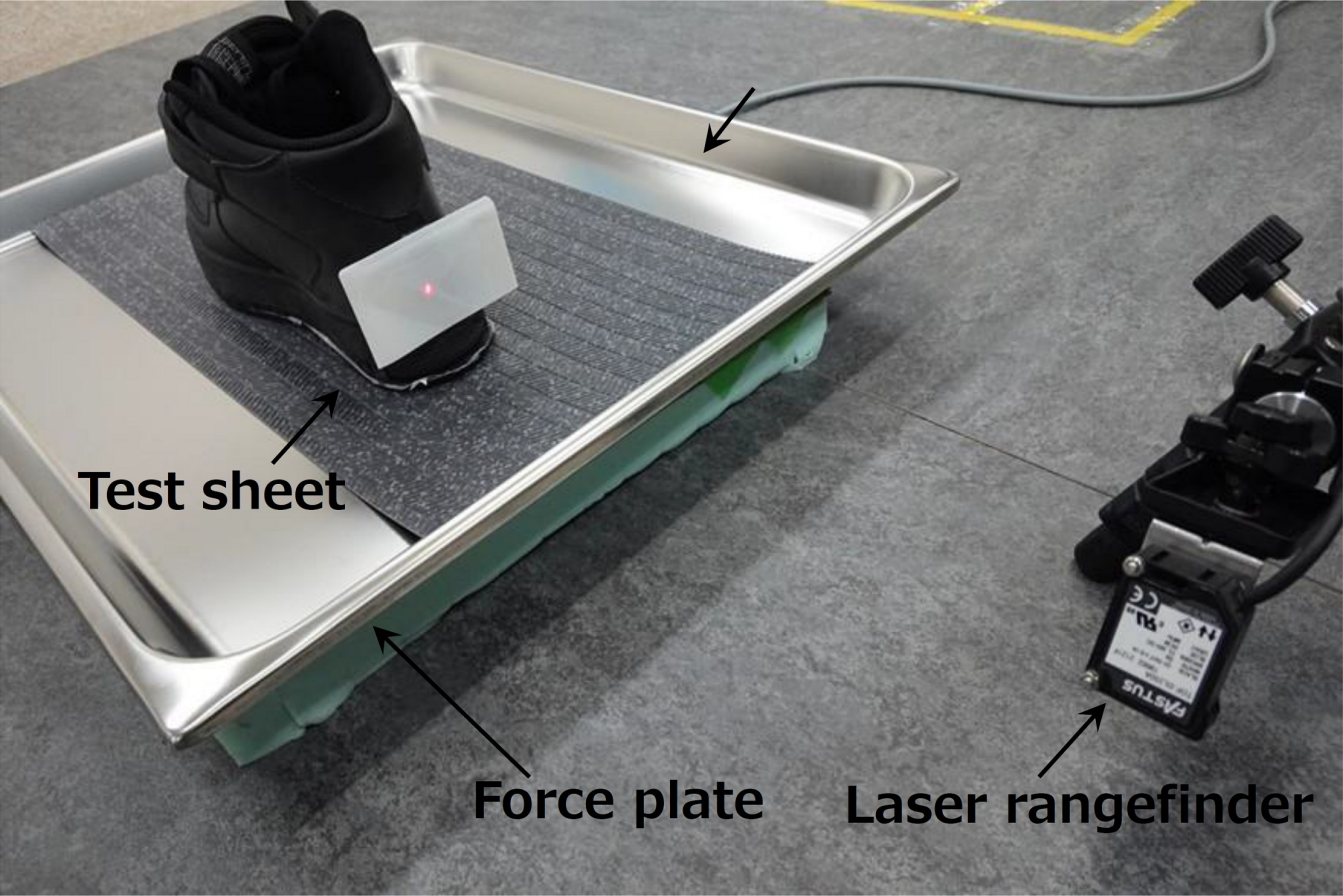
Photo of experimental setup.

The experimental setup used in the present study included a stainless-steel vat (650 × 530 × 40 mm), force plate (9286AA, Kistler Japan Co. Ltd, Japan), laser rangefinder (TOF-DL250A, Optex Group Co. Ltd, Japan), and personal computer, as shown in Fig 3. The sampling frequency for the force plate and laser rangefinder data was 1 kHz. The force plate was used to measure the ground reaction force and global position of the center of pressure (COP) (600 × 400 mm). The stainless-steel vat was attached to the force plate to prevent oil splashing during foot rubbing. The test sheets were attached to the stainless-steel vat. The same 90 wt% glycerol solution that was used in the mechanical friction tests was spread over the surface of the test sheet at approximately 200 g/m^2^. The spread amount was only at expected level, which was not strict. The laser rangefinder measured the movement of the heel of the test footwear to calculate its sliding velocity. The x-axis was set to the right side relative to the sliding direction, the y-axis pointed in the forward sliding direction, and the z-axis was set in the vertical direction normal to the force plate.

**Fig 4:**
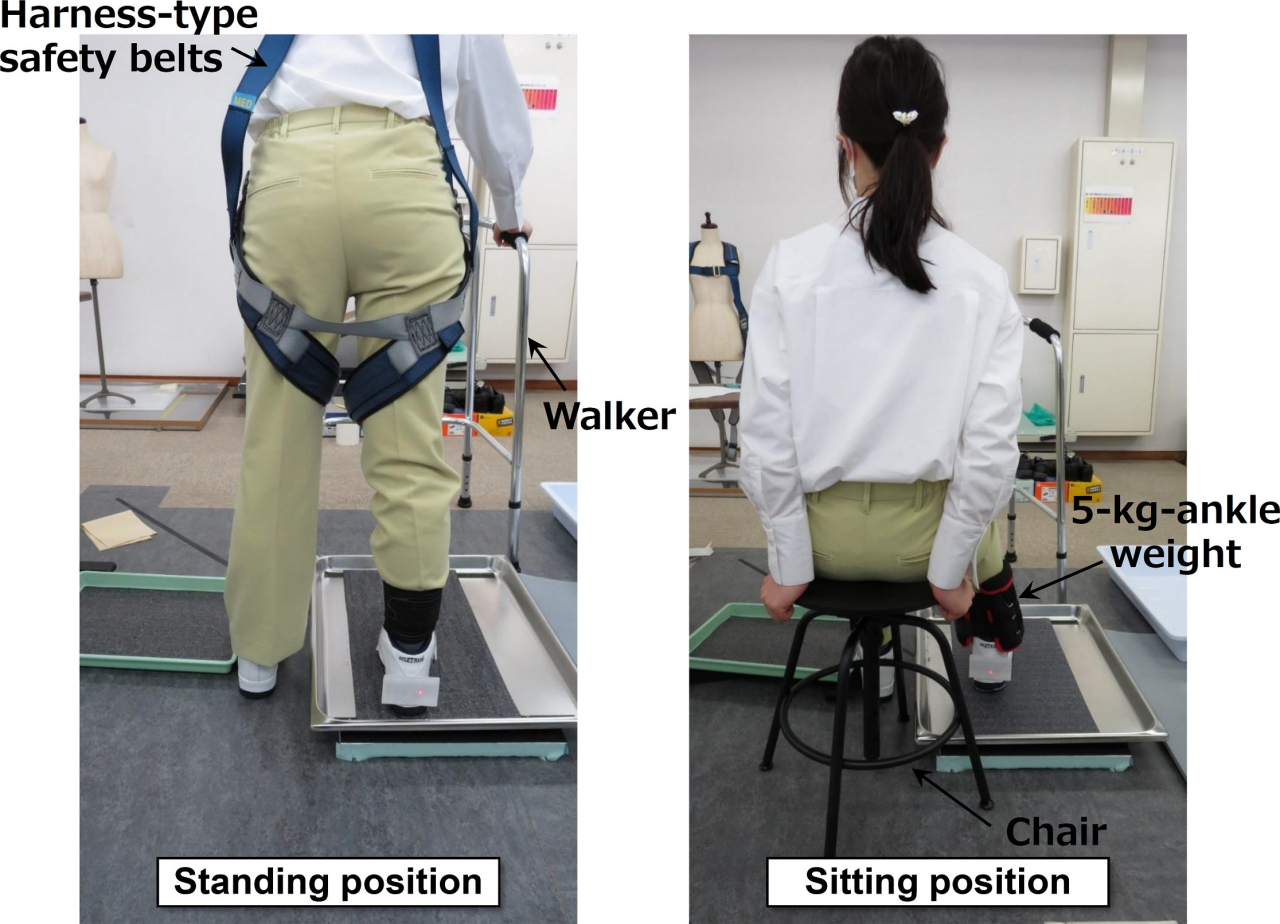
Photos of participants in standing and sitting positions.

The participants were instructed to rub their feet with eyes open in two different testing positions, standing and sitting as shown in Fig 4. They first conducted a series of tests in either the standing or sitting position throughout, and then finished those in the other position. As a safety consideration when conducting the experiment in the standing position, the participants were asked to wear harness-type safety belts attached to a rope fixed to a rail suspended from the ceiling while standing. Furthermore, the participants were tested in the standing position using a stainless-steel walker without casters. They were asked to rub their right foot against the oil-lubricated sheet once in the forward direction. The loading force and rubbing speed were arbitrary, depending on the individual. The participants were asked to keep the foot contact angle zero against the test sheet during sliding. After rubbing once, they gave two sensory scores for the slip resistance, including the slip onset and during sliding, using a visual analog scale (VAS) [25]. The full length of the VAS was 100 mm. The minimum score was zero at the left end, and the maximum was 100 at the right end. When the participants felt a small slip resistance, they gave a low score. The participants could mark on the VAS. In the sitting position, the participants were asked to sit on a height-adjustable chair without casters. They were also asked to wear a 5 kg weight on their right ankle instead of the harness-type safety belt to complement the relatively small vertical force. Before the main tests, preliminary tests confirmed that the vertical forces with the weight when sitting were similar to those when standing without any weight. Furthermore, we also learned that a feeling of strangeness appeared when rubbing the foot with an ankle weight larger than 5 kg. The participants gave sensory scores in the sitting position using the same VAS as used in the standing position test. The position that was tested first was random for the individual, with seven participants starting with the standing position.

The presentation sequence of the test sheets is described below. First, to determine the feeling of the maximum sensory score, we asked each participant to rub their foot on a dry stainless-steel vat, which showed the maximum SCOF and DCOF values compared with all the sheets when conducting the mechanical friction measurements, as shown in Table 1. The dry stainless-steel vat might have the highest value of sensory score. Second, to determine the minimum sensory score, we asked each participant to rub their foot on a glycerol-solution-lubricated stainless-steel vat, which showed the minimum SCOF and DCOF values. The oil-lubricated stainless-steel vat might have the smallest value of sensory score. We then randomly presented nine test sheets, with each sheet tested three times. Therefore, the total number of trials was 27 for each position, except for the dry and oil-lubricated stainless-steel vats.

### 2.4. Data analysis

**Fig 5:**
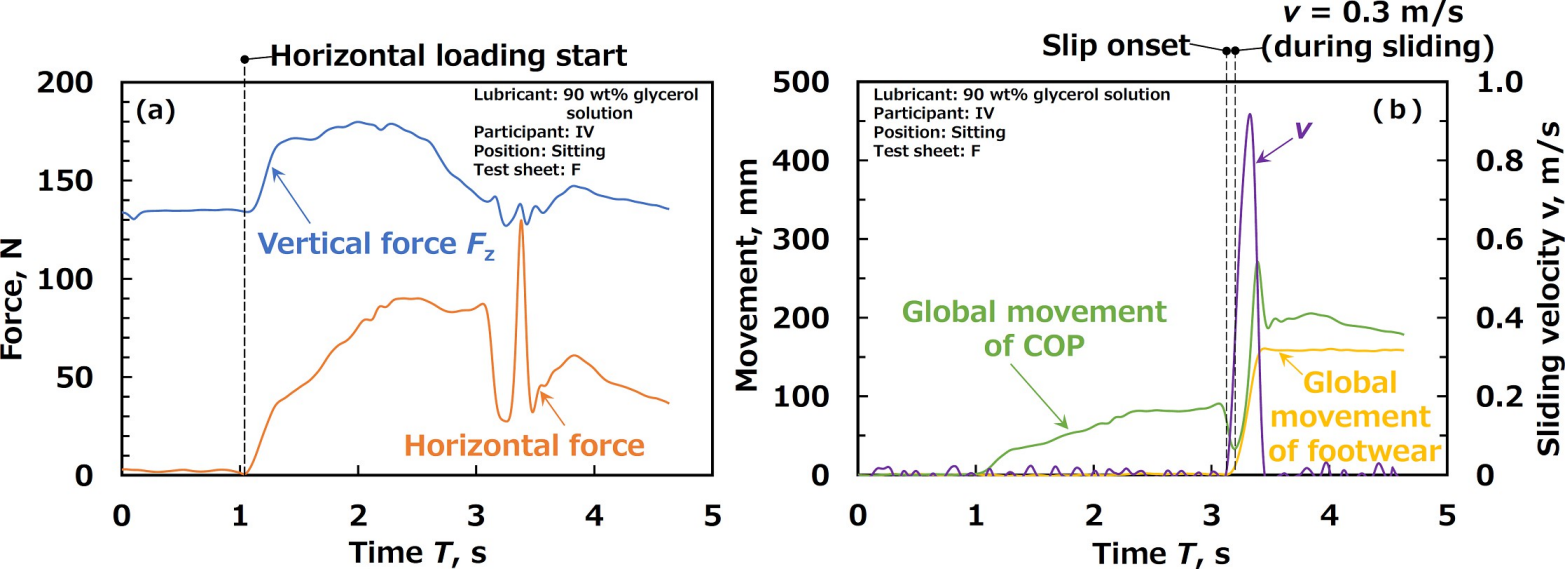
Examples of data obtained from force plate and laser rangefinder regarding (a) forces and (b) movements and velocity.

The friction force was investigated to examine whether this value correlated with the slip resistance sensory scores. The friction force can be defined as the magnitude of the horizontal reaction force (Fig 5A). The vertical force was also investigated to examine whether that its values differed between the standing and sitting positions. The COF was calculated by dividing the friction force by the vertical force to compare its tendency with that of the mechanical friction tests. The sliding velocity of the footwear was calculated from the difference between the global movements of the footwear at each sampling time (Fig 5B). The slip onset time was defined as the point just before the sliding velocity began to increase from zero. In addition, the time during sliding was defined as the period when the sliding velocity was 0.3 m/s in the acceleration period. The value of 0.3 m/s was taken from the Japanese Industrial Standard [24]. The suffix numbers 1, 2, 4, and 5 in the variables indicate the conditions of slip onset while standing, sliding while standing, slip onset while sitting, and sliding while sitting, respectively. In this study, we attempted to define the horizontal loading start time. The start of horizontal loading was defined as the time when the rate of increase in horizontal reaction force in y-axis exceeded 50 N/s. Fig 5A shows an example of the start of horizontal loading. This threshold was sufficiently greater than the fluctuations in the loading speed when a participant simply placed their foot on the test sheet. The suffix numbers 0 and 3 in the variables indicate the conditions at the loading start for standing and standing, respectively.

We also attempted to define the local movement of the COP within the participant’s sole. We could determine the global movement of the COP from the initial position and the global movement of the footwear from the initial position using the force plate and laser rangefinder, respectively (Fig 5B). Therefore, the local movement of the COP within the sole could be calculated as the difference when the global movement of the footwear was subtracted from the global movement of the COP. In this context, the local movement of the COP within the sole should have been zero when the two devices began recording. The distance between the two devices was not constant in order to adjust them according to the participant’s motion; therefore, the initial (recording start) position of the local COP within the sole, which was usually based on the heel, was unclear. The value of the local movement of the COP within the sole could be just described as the variation from the initial position.

All the data obtained were low-pass filtered with a cut-off frequency of 5 Hz using a fourth-order, zero-lag, Butterworth filter. The cut-off frequency was selected based on the literature [26].

Sensory scores were calculated from the length of the marked position on the VAS from the left end, which was zero. The measured length was divided by the full length of the scale (100 mm) and multiplied by 100. Therefore, the sensory scores were represented as percentages.

### 2.5. Statistical analysis

All statistical analyses were performed with EZR (version 1.55) [27], which is for R. More precisely, it is a modified version of R commander designed to add statistical functions frequently used in biostatistics.

For observing the tendency of linear correlations between the friction force and sensory score, the Pearson correlation coefficients were used. To increase the strength of the correlation, averaging was conducted for the same test sheet, which was presented three times. We set the criterion to a correlation coefficient of 0.74, which corresponded to a power value (1−β) of more than 0.8 for a statistical test when the significance level (α) was 0.05, and the sample size was nine under a two-tailed test. We could consider a participant who showed a correlation coefficient greater than 0.74 to be a person with friction perception capability.

When sample sizes are not sufficient to consider them as normally distributed data, we used non-parametric tests. In particular, the Wilcoxon signed-rank tests with a one-tailed distribution were used for paired data. For non-paired and non-parametric data, the Mann-Whitney’s U test s with a one-tailed distribution were selected.

Receiver operatorating characteristic (ROC) analysis [28] were used to determine a hazard detection score. Here, the object variable is a binarized COF, and the independent variable is the sensory score. The binarized COFs, for example 0 and 1, indicates whether the COFs exceeds 0.2 or not. The value of 0.2 for the COF is commonly used as a threshold to determine whether safety shoes have slip resistance [29]. This means that an interface between a flooring and shoe with a COF of less than 0.2 has a slip-induced fall risk. In the ROC curves, the sensitivities corresponded to a true positive rate to correctly detect a COF of less than 0.2. The specificity corresponded to a true negative rate to correctly detect a COF of more than 0.2. Areas under the curve (AUCs) were used as a predictive ability of the ROC curves. In general, an AUC value of more than 0.9 indicates high accuracy of the ROC curve. An optimal cut-off point, which optimally divides positive (a COF of less than 0.2) and negative (a COF of more than 0.2), was obtained from the point on the ROC curve closest to (1, 1 (specificity, sensitivity)). The cut-off points corresponded to critical values in the slip resistance score. In that kind of meaning, the critical slip resistance scores using a binarized COF threshold of 0.2 could divide a workplace into safe and hazardous. In particular, we defined the critical slip resistance score using a binarized COF threshold of 0.2 as a hazard detection score. In addition, the ROC analysis for binarized COFs by a value of 0.3, 0.4, and 0.5 were also conducted to see the effect of those on the ROC curves.

## 3. Results

### 3.1. Relationship between friction force and sensory score

**Fig 6:**
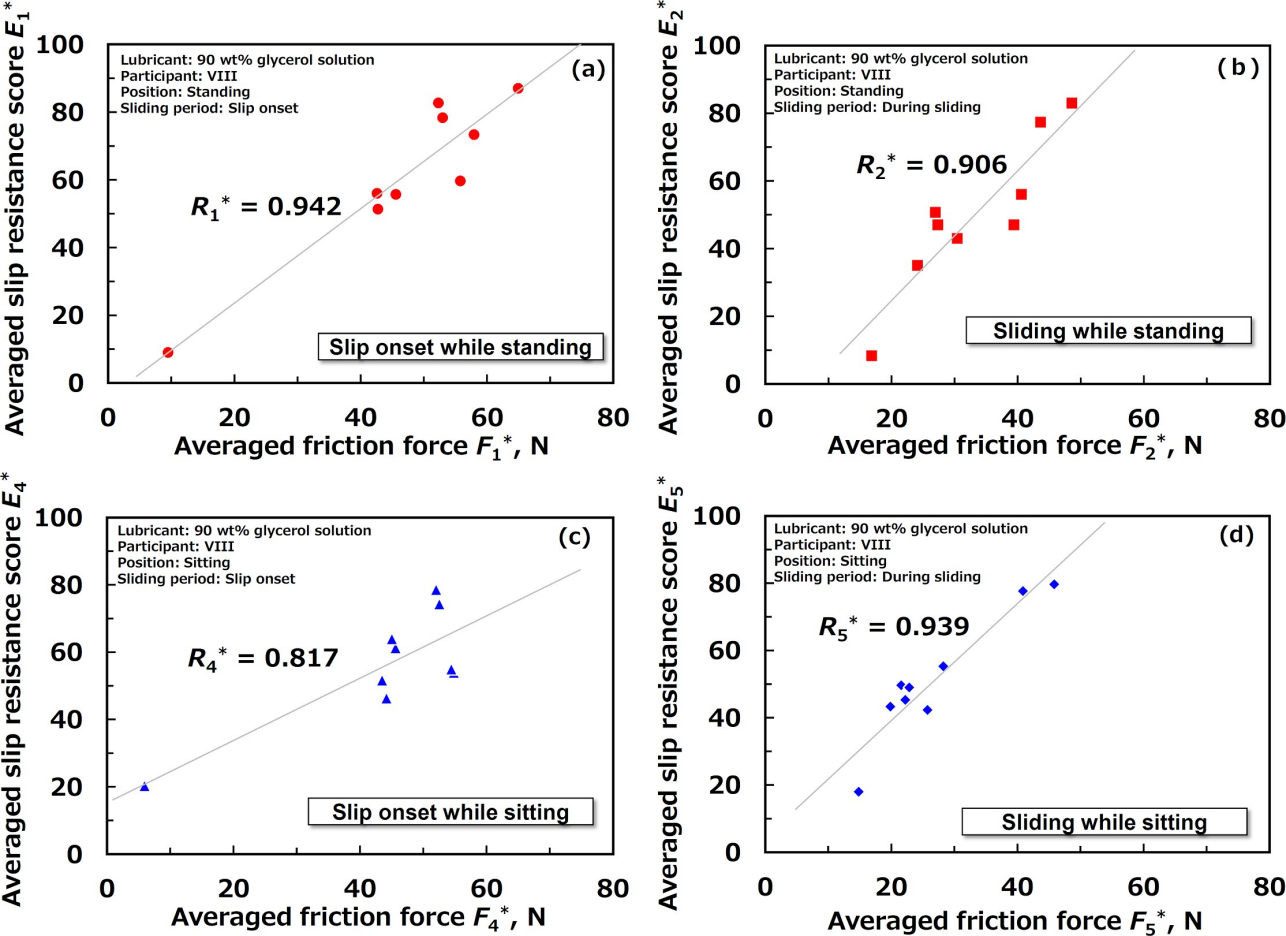
Relationship between friction forces and slip-resistance scores after averaging for participant VIII (a) at slip onset while standing, (b) during sliding while standing, (c) slip onset while sitting, and (d) during sliding while sitting.

Fig 6 shows the relationship between the friction force and slip resistance scores after averaging for participant VIII. These figures originally had error bars regarding the standard deviation; however, they were removed because of visibility. As shown in Fig 6, under either condition, the slip resistance score for participant VIII was linearly correlated to the friction force, with a correlation coefficient of greater than 0.74. It can be said that participant VIII could perceive the slip resistance with considerable accuracy, irrespective of the test position and sliding period.

It is well known that a subjective sensation is proportional to the logarithm of the stimulus intensity based on Fechner’s law [30, 31]. However, this leaves much room for discussion when applying the Weber–Fechner law to unexplained human perceptions [31, 32]. In addition, the magnitude range of the friction force in the present study was several times smaller than that in the case of representative perceived intensities following the law. For example, the intensity of the perceived loudness or brightness can vary by thousands of times or more. On the other hand, it is a fact that the numerical perceptions of Western children obey the Weber-Fechner law, but a shift from logarithm to linear perception occurs later in development as a result of maturity or scholarization [30, 33]. Hence, we assumed that in a narrow range of friction forces used to determine the stimulus intensity, the sensory scores would be linearly correlated with the friction forces of the participants who could adequately perceive it.

Table 2 lists the Pearson correlation coefficients between the friction force and sensory score for each participant after averaging. As shown in Table 2, the numbers of participants with friction perception capability were 9, 9, 8, and 7 out of 15 at slip onset while standing, sliding while standing, slip onset while sitting, and sliding while sitting, respectively. Almost half or more than half of the participants could adequately perceive the slip resistance using this method without any education about the perception.

**Table 2:** Correlation coefficients for each participant after averaging.

Participant	Correlation coefficient			
	Standing position		Sitting position	
	Slip onset *R*_1_*	Sliding *R*_2_*	Slip onset *R*_4_*	Sliding *R*_5_*
I	0.605	0.803	0.611	0.653
II	0.890	0.918	0.443	0.412
III	0.772	0.659	0.884	0.894
IV	0.782	0.652	0.900	0.900
V	0.565	0.927	0.717	0.677
VI	0.861	0.845	0.837	0.748
VII	0.907	0.752	0.908	0.838
VIII	0.942	0.906	0.817	0.939
IX	0.537	0.837	0.509	0.890
X	0.666	0.344	0.910	0.648
XI	0.820	0.533	0.825	0.396
XII	0.595	0.949	0.705	0.661
XIII	0.898	0.560	0.719	0.840
XIV	0.406	0.735	0.404	0.162
XV	0.848	0.762	0.769	-0.481

To determine whether the standing or sitting position was superior, the Wilcoxon signed-rank tests with a one-tailed distribution of the correlation coefficient (*R*) were conducted for both the slip onset and during sliding. There was no significant difference between the standing and sitting positions, irrespective of the slip onset and during sliding (p = 0.489 and 0.180, respectively). At the present stage, it cannot be clearly concluded which is a better way to perceive slip resistance.

### 3.2. ROC analysis to determine threshold

**Fig 7:**
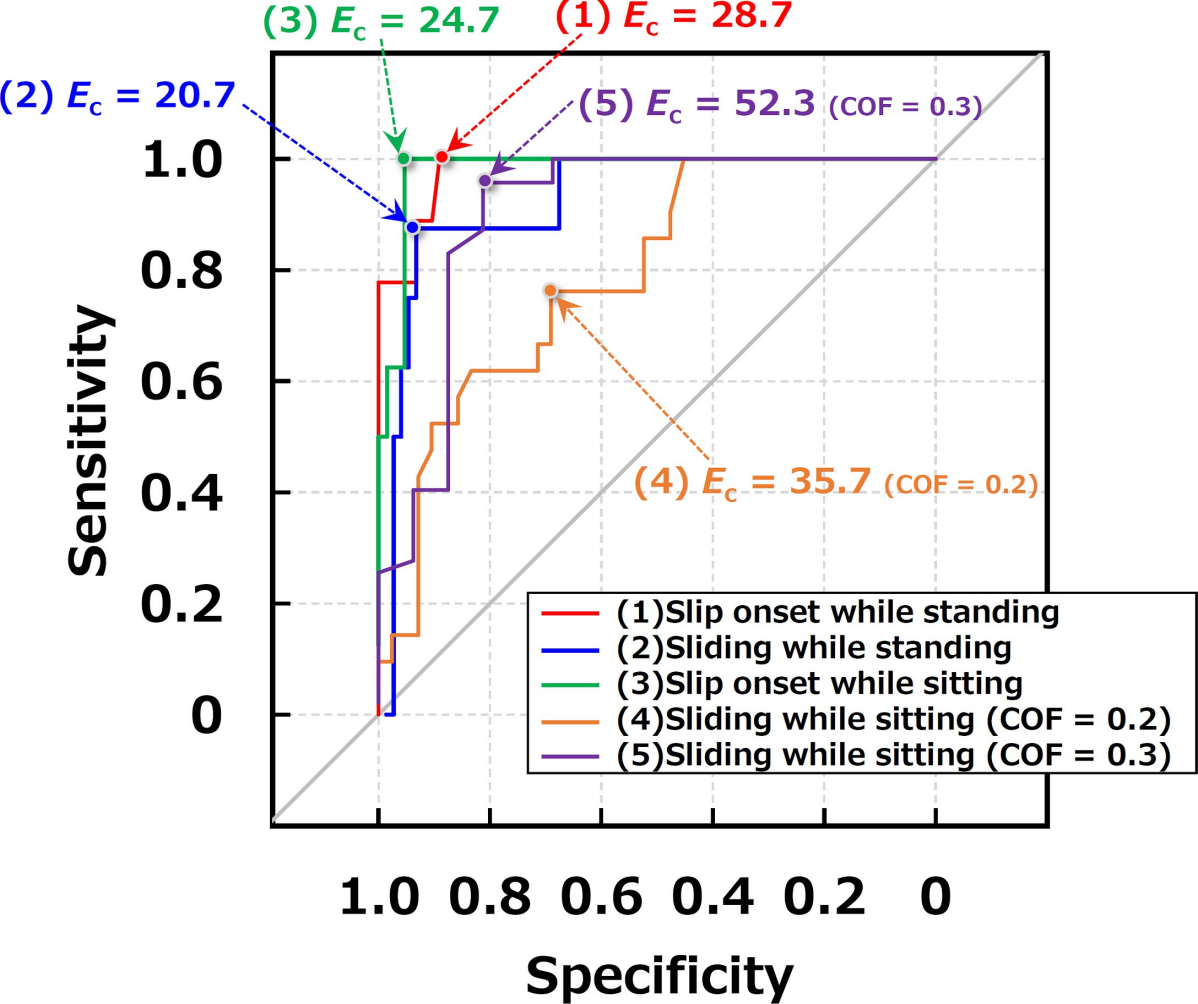
ROC curves for participants with the capability of perceiving friction.

Fig 7 shows the ROC curves for participants with the capability of perceiving friction. In addition, Table 3 lists the details of these ROC analysis. The hazard detection scores were 28.7, 20.7, 24.7, and 35.7 for slip onset while standing, sliding while standing, slip onset while sitting, and sliding while sitting, respectively. However, The AUC for sliding while sitting was 0.791, which meant moderate accuracy. From the standpoint of safety management, the determined hazardous area should have a wide margin. Therefore, we should use the critical slip resistance score using a binarized COF threshold of 0.3 (52.3) as a hazard detection score for sliding while sitting, where the AUC was 0.902. Thus, a hazardous workplace with a high slip-induced fall risk could be revealed using the suggested foot rubbing method and threshold scores. From the viewpoint of hazard detection accuracies, the standing position was a better way than the sitting although limited to participants with the capability of perceiving friction.

**Table 3:** Details of ROC analysis associated with Fig 7.

Condition	Participant with capability of perceiving friction	Threshold of binarized COF	Critical value in slip resistance score (specificity, sensitivity)	AUC (95% confidence interval)
Slip onset while standing	II, III, IV, VI, VII, VIII, XI, XIII, XV (9 participants)	0.2	28.7 (0.890, 1.000)	0.981 (0.952–1.000)
Sliding while standing	I, II, V, VI, VII, VIII, IX, XII, XV (9 participants)	0.2	20.7 (0.932, 0.875)	0.926 (0.844–1.000)
Slip onset while sitting	III, IV, VI, VII, VIII, X, XI, XV (8 participants)	0.2	24.7 (0.953, 1.000)	0.980 (0.953–1.000)
Sliding while sitting	III, IV, VI, VII, VIII, IX, XIII (7 participants)	0.2	35.7 (0.690, 0.762)	0.791 (0.678–0.905)
		0.3	52.3 (0.812, 0.957)	0.902 (0.789–1.000)

Here, we did not investigate the relationship between the sensory score obtained in this study and “real” slip-induced risk, thus, it is unclear whether the proposed method can find a hazardous area with a “real” high slip-induced risk. In the future works, we have to verify the reliability of the hazard detection scores at real workplace to reduce the occupational fall accidents.

## 4. Discussion

### 4.1. Vertical force

Table 4 lists the mean vertical forces at slip onset for each participant. The individuals had a wide range of values. Some participants could not reach the critical load of 159–315 N, which was 30% of their body weight. This was because the participants were instructed to rub their foot using an arbitrary loading force and rubbing speed. Because the mean vertical force did not depend on the gender, body weight, and age of the participants, it depended on the habitual force that they used when rubbing their foot. Interestingly, there was almost no difference between the standing and sitting positions except for the participant II, VII, and XII. The ankle weight used in the sitting position played a role in this context. On the other hand, the standard deviations for the standing position were larger than those for the sitting position. It was assumed that because the participants grasped the walker’s handle for safety, they could release a vertical force in the standing position more easily than in the sitting position, which restricted the vertical movement of the participant’s foot. Conversely, the sitting position allowed them to maintain a stable vertical force while rubbing their foot.

**Table 4:** Mean vertical forces at slip onset for each participant.

Participant	Standing position		Sitting position	
	Mean vertical force *F*_z1_, N	(S.D.)	Mean vertical force *F*_z4_, N	(S.D.)
I	89	50	79	17
II	242	55	127	20
III	142	37	143	24
IV	108	37	155	21
V	179	80	156	13
VI	223	57	196	22
VII	369	53	206	31
VIII	96	42	114	13
IX	128	61	133	22
X	100	22	122	20
XI	148	97	191	19
XII	151	64	82	18
XIII	97	39	107	14
XIV	79	56	97	20
XV	110	63	94	17

### 4.2. SCOF and DCOF

**Fig 8:**
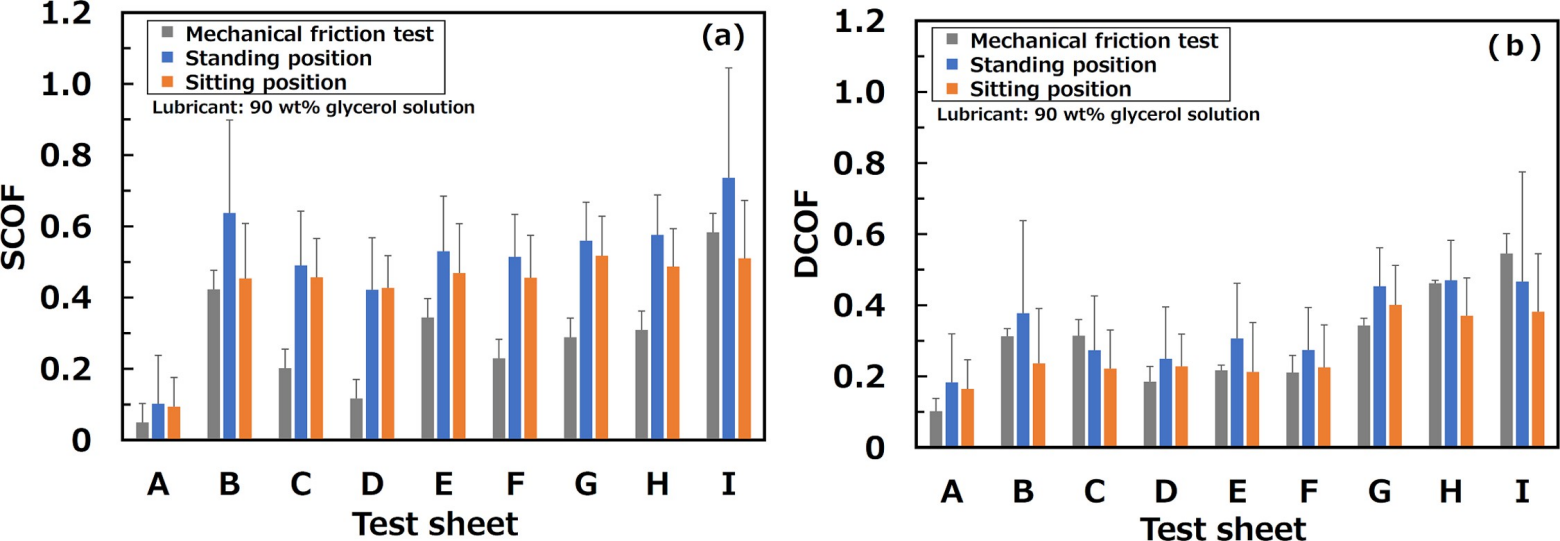
Comparison of COFs between mechanical friction test and foot rubbing test: (a) SCOFs and (b) DCOFs.

Table 5 and Fig 8 show the mean SCOFs and DCOFs between test sheets and test shoes found in the foot rubbing tests for all the participants’ trials and a comparison of the COFs between the mechanical friction test and foot rubbing test. The error bars indicate the standard deviation of the COF among the trials. There was almost no difference in the tendency of the order of the SCOFs between the mechanical friction test and foot rubbing in standing position. However, the magnitudes of the SCOF for in standing position were greater than those for the mechanical friction test, except for sheet A. This was because the vertical forces in the standing position were slightly smaller than those in the mechanical friction tests. However, the order of the SCOFs in the sitting position differed from the others. This may have been because the main contact area in the sole could have differed from that in the standing position (for example, one the side of the heel). The DCOFs in both the standing and sitting positions were smaller than the SCOFs. Therefore, the DCOFs were almost the same as those of the mechanical friction and foot rubbing tests. It can be said that there was no difference in the intrusion of the glycerol solution into the flooring/shoe interface between the mechanical friction tests and foot rubbing tests during sliding after slip onset.

**Table 5:** Mean SCOFs and DCOFs between test sheets and test shoe for foot rubbing tests. (a) Standing position. (b) Sitting position.

Sheet	Mean SCOF	(S.D.)	Mean DCOF	(S.D.)
(a)				
A	0.101	0.137	0.183	0.102
B	0.637	0.261	0.377	0.165
C	0.490	0.152	0.274	0.082
D	0.422	0.146	0.249	0.052
E	0.530	0.155	0.306	0.116
F	0.514	0.120	0.274	0.069
G	0.559	0.109	0.453	0.096
H	0.576	0.113	0.470	0.223
I	0.736	0.308	0.467	0.199
(b)				
A	0.094	0.082	0.165	0.058
B	0.454	0.154	0.236	0.073
C	0.457	0.109	0.222	0.039
D	0.427	0.091	0.228	0.066
E	0.469	0.139	0.212	0.070
F	0.455	0.119	0.225	0.062
G	0.517	0.111	0.401	0.075
H	0.487	0.106	0.370	0.060
I	0.510	0.163	0.382	0.152

### 4.3. Comparison of correlation coefficient between before and after averaging

For an instant evaluation in workplace, trials should be conducted with a small number of times. To consider the availability of less trials, the effect of data averaging should be discussed. If the correlation coefficient does not change before and after averaging, we can adopt a single trial test for each flooring in a workplace. Before averaging, we had 27 trials for each participant. According to Cohen [34], a large effect size is defined as a correlation coefficient that exceeds 0.5. This was sufficient for a power value (1−β) of more than 0.8 for a statistical test when the significance level (α) was 0.05, and the sample size was 27 under a two-tailed test. Table 6 shows the Pearson correlation coefficients for each participant before averaging. The numbers of participants, who showed a correlation coefficient greater than 0.5, were 12, 10, 12, and 11 out of 15 for slip onset while standing, sliding while standing, slip onset while sitting, and sliding while sitting, respectively. Although these seem to be higher percentages compared to the averaged data, the criterion of a correlation coefficient of 0.5 is commonly considered to be a moderately strong correlation. For almost all the participants, the correlation coefficients in Table 2 after averaging are greater than those in Table 6 before averaging. This was because changeable interface conditions between the oil-lubricated sheet and shoe easily led to a fluctuation in the friction force in some extent, and the forward one-time foot rubbing was too momentary to finely perceive the friction force. The multiple trials and averaging were a valid way to increase the reliability of the friction perception for the forward one-time foot rubbing. Round-trip rubbing or long-term rubbing was also suggested as a valid method.

**Table 6:** Correlation coefficients for each participant before averaging.

Participant	Standing position		Sitting position	
	Correlation coefficient for slip onset *R*_1_	Correlation coefficient for during sliding *R*_2_	Correlation coefficient for slip onset *R*_4_	Correlation coefficient for during sliding *R*_5_
I	0.441	0.499	0.480	0.560
II	0.579	0.855	0.351	0.530
III	0.717	0.552	0.824	0.784
IV	0.748	0.511	0.705	0.718
V	0.546	0.746	0.557	0.579
VI	0.692	0.628	0.757	0.649
VII	0.820	0.638	0.885	0.765
VIII	0.913	0.764	0.638	0.826
IX	0.566	0.833	0.562	0.759
X	0.497	0.318	0.758	0.417
XI	0.686	0.485	0.752	0.362
XII	0.536	0.824	0.532	0.507
XIII	0.783	0.480	0.633	0.734
XIV	0.265	-0.031	0.352	-0.023
XV	0.791	0.679	0.707	-0.403

### 4.4. Test accuracy of ROC analysis by changing threshold

Table 7 shows the details of the ROC analysis by changing threshold of binarized COF from 0.3 to 0.5. Compared to Table 3, the AUCs in a binarized COF threshold of 0.2 exhibit the highest value among those in the other thresholds for slip onset, irrespective of standing and sitting positions. Furthermore, the AUCs decreases with an increase in the binarized COF threshold. This meant that the participants could easily perceive low friction at slip onset because drastic changes of foot movement in the moment. The AUC in a threshold of 0.2 also shows the highest value for sliding while standing. The low DCOFs in the standing position could lead to balance losses of lower limbs or whole bodies, resulting in strong impression in a negative way. In contrast, the AUC in a threshold of 0.5 shows the highest value for sliding while sitting. In sitting position, the sliding distance of the foot was restricted compared to the standing because the thighs were on the chair. There is a possibility of unintentional stop of the foot movement in sitting position at even low DCOFs. Therefore, easier stop of the foot movement might result in strong impression in a positive way in sitting positions while sliding. From the viewpoint of hazard detection accuracies, the standing position was again a better way than the sitting for the participants with the capability of perceiving friction.

**Table 7:** Details of ROC analysis by changing threshold of binarized COF from 0.3 to 0.5.

Condition	Participant with capability of perceiving friction	Threshold of binarized COF	Critical value in slip resistance score (specificity, sensitivity)	AUC (95% confidence interval)
Slip onset while standing	II, III, IV, VI, VII, VIII, XI, XIII, XV (9 participants)	0.3	28.7 (0.914, 0.917)	0.979 (0.952–1.000)
		0.4	45.0 (0.776, 0.792)	0.860 (0.773–0.947)
		0.5	46.7 (0.750, 0.524)	0.688 (0.559–0.817)
Sliding while standing	I, II, V, VI, VII, VIII, IX, XII, XV (9 participants)	0.3	37.3 (0.814, 0.692)	0.826 (0.739–0.912)
		0.4	54.3 (0.857, 0.852)	0.904 (0.824–0.984)
		0.5	56.3 (0.778, 0.753)	0.825 (0.697–0.953)
Slip onset while sitting	III, IV, VI, VII, VIII, X, XI, XV (8 participants)	0.3	33.3 (0.871, 1.000)	0.962 (0.921–1.000)
		0.4	47.7 (0.809, 0.880)	0.885 (0.796–0.974)
		0.5	58.3 (0.889, 0.759)	0.876 (0.796–0.955)
Sliding while sitting	III, IV, VI, VII, VIII, IX, XIII (7 participants)	0.3	52.3 (0.812, 0.957)	0.902 (0.789–1.000)
		0.4	53.7 (1.000, 0.831)	0.911 (0.827–0.995)
		0.5	65.0 (1.000, 0.951)	0.959 (0.912–1.000)

### 4.5. Exploration of factors to determine friction perception capability

In the present study, almost half or more than half of the participants could perceive slip resistance adequately using the proposed method without any education about perception. Ideally, all of the participants should have been able to adequately perceive the slip resistance. To improve this method in the future, it will be necessary to determine what caused the difference in the participant’s slip perceptions.

We focused on how to apply forces that might depend on the individual. In particular, the variations in the vertical force and movement of the COP within the sole were calculated. As a hypothesis, we investigated whether smaller variations and movements might result in a higher slip perception sensitivity. As a variation of the vertical force, the vertical force at the start of horizontal loading (*F*_z0,_ *F*_z3_) was subtracted from that at the slip onset (*F*_z1,_ *F*_z4_) or during sliding (*F*_z2,_ *F*_z5_). For the movement of the COP within the sole, the local movement of the COP, as previously mentioned, was calculated from the horizontal loading start to the slip onset or during sliding. Table 8 lists the mean variations in the vertical forces and mean local movements of the COP within the sole for each participant. A positive value for the mean variation in the vertical force indicates an increase in the vertical force. A positive value for the mean movement of the COP indicates a forward movement along the sliding direction. We also conducted the Mann-Whitney’s U test s with a one-tailed distribution between the referenced participants and others regarding these two parameters, as listed in Table 9. This test was used because the comparison of data between the referenced participants and others was unpaired. In the standing position, there was a significant difference in the movement of the COP at slip onset when the significance level was 5%. Briefly, a small movement of the COP after the loading start would increase the slip perception sensitivity at slip onset in standing position. As mentioned above, the participant could easily change the vertical load in the standing position. Thus, the area of the sole that was perceived would affect the sensitivity of slip perception rather than how it was loaded. Unfortunately, there was no significant difference during sliding in standing position, which meant that other factors existed. For the sitting position, there was a significant difference in the variation of the vertical force, irrespective of the slip onset and during sliding. The variations in the vertical force for the referenced participants with a positive value were significantly greater than those for the others with negative values. Thus, increasing the vertical load after the loading start would increase the slip perception sensitivity in the standing position rather than releasing the vertical force. A future task could involve determining whether these two factors, a small movement of the COP while standing and an increase in the vertical load while sitting, could be used educationally to improve slip perception.

**Table 8:** Mean variation of vertical forces and mean local movements of COP within sole. (a) Slip onset while standing. (b) Sliding while standing. (c) Slip onset while sitting. (d) Sliding while sitting.

Participant with capability of perceiving friction (R* ≥ 0.74)			Other participant (R* < 0.74)		
Participant	Mean variation of vertical force *F*_z2-_ *F*_z0, N_	Mean movement of COP within the sole, mm	Participant	Mean variation of vertical force *F*_z2-_ *F*_z0, N_	Mean movement of COP within the sole, mm
(a)					
II	-151	86	I	-178	85
III	-111	84	V	-251	142
IV	-153	81	IX	-186	127
VI	-189	89	X	-134	111
VII	-17	99	XII	-259	113
VIII	-187	103	XIV	-173	125
XI	-266	64			
XIII	-170	107			
XV	-174	83			
Average	-158	88	Average	-197	117
(S.D.)	63	12	(S.D.)	44	18
(b)					
I	-162	101	III	-107	53
II	-146	59	IV	-112	33
V	-280	140	X	-135	58
VI	-144	44	XI	-253	19
VII	-35	89	XIII	-174	65
VIII	-184	73	XIV	-209	142
IX	-204	119			
XII	-265	66			
XV	-160	45			
Average	-176	82	Average	-165	62
(S.D.)	68	31	(S.D.)	53	39
(c)					
III	13	45	I	-46	31
IV	22	63	II	-13	54
VI	35	31	V	-15	77
VII	39	40	IX	-10	64
VIII	-1	47	XII	-8	81
X	-16	84	XIII	-17	74
XI	17	32	XIV	-29	75
XV	-30	46			
Average	10	48	Average	-20	65
(S.D.)	22	16	(S.D.)	12	16
(d)					
III	12	5	I	-38	5
IV	18	29	II	-19	18
VI	39	-8	V	-7	40
VII	44	1	X	-17	33
VIII	5	5	XI	16	-8
IX	-9	32	XII	-16	18
XIII	-17	41	XIV	-31	72
			XV	-22	2
Average	13	15	Average	-17	22
(S.D.)	21	17	(S.D.)	15	24

**Table 9:** Probability values in Mann-Whitney’s U test between participants with friction perception capability and others.

Condition	For mean variation of vertical force	For mean movement of COP within sole
Slip onset while standing	0.194	0.004
Sliding while standing	0.736	0.909
Slip onset while sitting	0.002	0.060
Sliding while sitting	0.007	0.306

### 4.6. Advantages of proposed foot rubbing evaluation method

Many studies used a five-point or seven-point, at most eleven-point scale for an evaluation of slipperiness. These ordinal scales are not suitable for a fine discrimination to determine a sensory threshold. In this study, we used an interval scale, VAS, that can determine a sensory threshold in detail.

A lot of researchers focused on the relation between a sensory score regarding slipperiness and mechanical friction property that was measured by a friction tester before or after the sensory test. In this case, the experimental conditions in sensory and friction tests may strictly differ. Therefore, they may observe the indirect relationship between the sensory score and friction property. On the other hand, for the foot rubbing evaluation method proposed in this study, the COFs were measured concurrently with the sensory evaluation. It contributed to the direct relationship, which led to more precise analysis.

## 5. Conclusions

A simple and inexpensive method to evaluate slip resistance that can be readily introduced into the workplace is required. In the present study, we investigated the relationship between a simple sensory evaluation of anti-slipperiness by foot rubbing in standing and sitting positions and the actual friction properties obtained with in situ measurements at slip onset and during sliding. Furthermore, the possibility of identifying a hazardous area with a high slip-induced fall risk was verified. An investigation was also conducted to determine whether the standing or sitting position was superior in term of the accuracy. The conclusions are summarized below.

1. The numbers of the participants with friction perception capability, who possessed a correlation coefficient of more than 0.74 between the friction force and sensory score, were 9, 9, 8, and 7 out of 15 at the slip onset while standing, sliding while standing, slip onset while sitting, and sliding while sitting, respectively.
2. The hazard detection scores that determined a hazardous area with a high slip-induced fall risk were obtained from the ROC curves for participants with friction perception capability. The scores were 28.7, 20.7, 24.7, and 52.3/100 for the slip onset while standing, sliding while standing, slip onset while sitting, and sliding while sitting, respectively.
3. The standing position was a better way than the sitting to determine a hazardous area with a high slip-induced fall risk although limited to participants with the capability of perceiving friction.

## Supporting information

S1 File Shibata et al data. (XLSX)
